# Contemporary Speech and Oral Language Care for Deaf and Hard-of-Hearing Children Using Hearing Devices

**DOI:** 10.3390/jcm9020378

**Published:** 2020-01-30

**Authors:** François Bergeron, Aurore Berland, Dominique Demers, Suzie Gobeil

**Affiliations:** 1 Department of Rehabilitation, Medical Faculty, Université Laval, QC G1V 0A6, Canada; dominique.demers.2@ulaval.ca; 2 Unité de Recherche Interdisciplinaire Octogone, EA4156, Laboratoire Cognition, Communication et Développement, Université Jean Jaurès, 31058 Toulouse, France; aurore.berland@hotmail.com; 3 CIUSSS Capitale Nationale, QC G1W 1P7, Canada; suzie.gobeil.ciussscn@ssss.gouv.qc.ca

**Keywords:** speech, language, care, deaf and hard of hearing, child

## Abstract

Contemporary speech and language interventions are not limited to disabilities but embrace the pragmatics of communication behaviors from the perspective of functional social participation. Accordingly, current speech and language therapies for deaf and hard-of-hearing children include a broad spectrum of approaches and techniques. This paper explores contemporary approaches and techniques for speech and oral language interventions for deaf and hard-of-hearing children using hearing devices, evidence of efficacy and how they are implemented in diverse clinical practices.

## 1. Introduction

The outcomes of hearing impairment in children are well documented. Indeed, many studies have shown that depriving auditory input, at any degree, can not only impede auditory perception, but also affect the development of the peripheral and central auditory pathways [1]. Because of an altered auditory input, speech [2] and oral language [3] development can be limited, which will impact oral communication skills [4] and, eventually, social participation [5] including academic achievement [6], involvement in leisure activities [7] and, eventually, access to the job market [8]. 

The efficacy of technological care on hearing impairment has extensively been shown; early hearing aids and cochlear implants can attenuate sensory deprivation [9], promote auditory [10], speech [11] and oral language [12] development and, ultimately, support social participation [13]. However, as suggested in the late 1980s by William House, “intervention in deafness is 10% hardware and 90% software”, emphasizing that technological care is only one part of the intervention, the most important part being the care of the individual. Indeed, despite the huge sophistication of modern hearing devices, none can replace a normal auditory system. Thus, complementary therapeutic care is still needed. 

**Figure 1:**
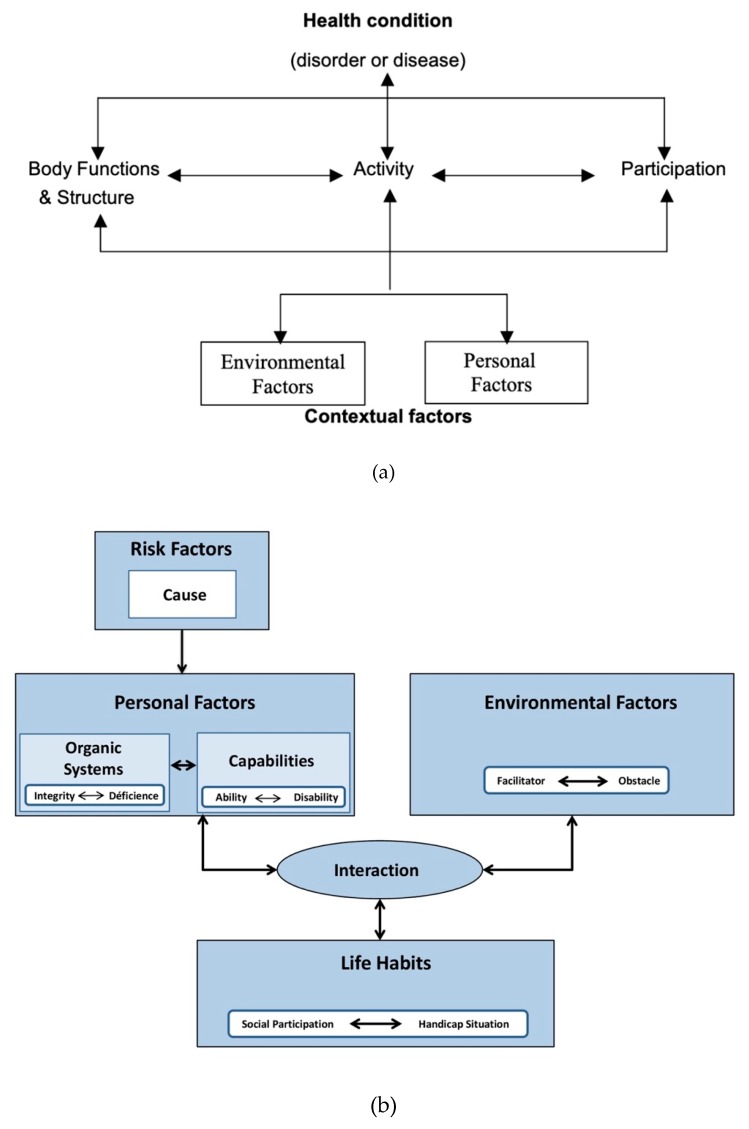
(**a**). The International Classification of Functioning, Disability and Health (ICF) model [14]. (**b**). Human Development Model—Disability Creation Process (HDM-DCP) RIPPH 1998 [15].

As stated before, social participation is an optimal objective of hearing health care; while intervention on the deficit, either medically of technologically, traditionally used to be considered as the way to restore (some) hearing and thus social participation, contemporary perspectives propose a more global approach to intervention. Both WHO’s Model of Functioning, Disability, and Health [14] and the Human Development Model of Disability Creation Process [15] suggest that disability emerges from the interaction between the individual and his environment in the context of his own life habits (Figure 1). Therefore, intervention should consider the individual’s deficit but also his incapacities, his environment and his life habits; this is especially true when communication is the core object of the intervention. 

The early days of speech and language intervention for the deaf and hard-of-hearing child were in line with the traditional linear approach to address disability as therapeutic approaches focused on training the incapacities with activities such as drills to push the emergence of an absent phoneme in speech or to fill holes in the child lexicon. However, soon, in accordance with the WHO/DCP disability models, clinicians realized that the scope of speech and language intervention could not be limited to incapacities but should embrace the pragmatics of communication behaviors from the perspective of functional social participation. Accordingly, current speech and language therapies for deaf and hard-of-hearing children are grounded on interactionist perspectives and thus include a broad spectrum of approaches and techniques. 

## 2. Contemporary Rehabilitation Approaches and Techniques

**Figure 2:**
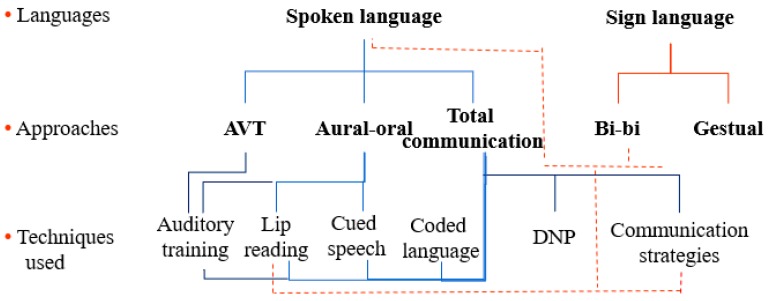
Relations between languages, approaches and techniques.

Communication is a key factor of social participation. Depending on many factors, such as associated disorders, age at implantation and duration of deafness, residual hearing before implantation, parents’ language and preferences or auditory skills level, two language options can be considered to support communication for the deaf and hard-of-hearing child [16,17] (Figure 2). The first option is spoken (oral) language. Since the implementation of newborn screening programs and early intervention with identified children, combined with the efficacy of contemporary technologies, oral language is the selected option for the vast majority of parents, largely because more than 90% of deaf children have hearing parents [18]. The other possible option is sign language. This alternative is generally preferred by deaf parents of deaf children or for children who do not have sufficient capacities to develop oral language despite the use of hearing devices, a typical situation when children were implanted after the sensitive period of auditory maturation. Notwithstanding the natural trend of parents to prefer the language alternative that reflect their own language, some question the extend of information that is presented to parents following the hearing deficit diagnosis; indeed, many parents wish that more information on communication mode alternatives and schooling options was available to support their decision [13].

### 2.1. Approaches

**Figure 3:**
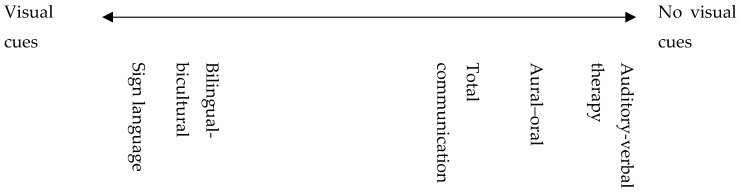
Approach continuum.

In order to help children develop their language skills, five main communication approaches are nowadays suggested to families [19]. These approaches can be viewed on a continuum from inclusion of “visual cues” to “no visual cues” (Figure 3).

#### 2.1.1. Auditory-Verbal Therapy (AVT)

This approach promotes the exclusive use of auditory skills to develop language abilities, the main objective being a seamless integration of the child in the community. In order to achieve this goal, access to visual cues, such as lipreading or facial expressions, is restricted during therapies. At home, parents must try to reduce as much as possible access to visual cues. AVT is heavily centered upon the parental implication in the rehabilitation process [20].

#### 2.1.2. Aural–Oral Communication

In this approach, language is developed from hearing capacities, but access to natural cues such as lipreading, natural gesture and facial expressions is allowed. In some cases, clinicians can use cued speech, in which sounds of speech are sign coded, to help children differentiate subtle aspects of speech [19].

#### 2.1.3. Total Communication (TC)

The main objective of TC is to install early communication between the child and his environment. As such, TC promotes oral language development through the use of many types of cues simultaneously: auditory input, natural cues, lip-reading cued speech but also natural and formal signs such as signed English or American Signed English (ASL). Thus, children and parents learn to use them, so that these cues can support both expressive and receptive language. This approach remains flexible, since the choice of cues depends on children’s needs and can vary over time (e.g., when the child becomes more fluent with oral language, the use of signs often decreases) [21].

#### 2.1.4. Bilingual-Bicultural (Bi-Bi)

This approach promotes the learning of both sign and oral languages (bilingual), as well as the integration in deaf and hearing cultures (bicultural). Sign language is learned as a primary language. Then, as a second language, the child learns oral language. Thus, both languages and both cultures are taught in schools using that approach. Learning oral language helps children to be integrated in society and supports reading/writing development. [19].

#### 2.1.5. Sign Language

This approach promotes the use of sign language (like ASL) for communication. As with oral language, sign language has its own grammar, vocabulary and expressions. It is based on the principle that since the child is deaf, his primary and most natural way to communicate is visual [19].

While some centers and/or professionals promote the exclusive implementation of one alternative over the others, others integrate the cohabitation of many.

### 2.2. Techniques

In connection with the different communication approaches, a range of techniques can be used by rehabilitation professionals in therapy. These therapeutic techniques aim the development of communications skills that will support each child’s social participation. 

#### 2.2.1. Auditory Training

As stated before, access to hearing devices such as hearing aids or cochlear implant is not sufficient for deaf children to develop oral communication abilities. An incomplete access to the sound environment limits the opportunities to naturally develop auditory skills (namely detection, discrimination, identification, recognition and comprehension). Auditory training is a technique that aims for the refinement of those skills and the maximal benefit of hearing devices through listening exercises [22,23]. Clinicians help the child to perceive, analyze and give some meaning to environmental and speech sounds. 

#### 2.2.2. Lip-Reading

In complement to auditory information, individuals commonly use visual cues to help understand speech. Lip-reading is thus a natural method, where mouth placement is used to support communication. The combination of auditory and visual cues is even more helpful for hearing impaired children, especially in noisy environments where auditory cues can be unclear. However, even if lipreading carries important on sound produced, this method cannot resolve situations where oral sounds are visually similar (e.g., /b/ and /p/). Even if all the children use lip-reading on a daily basis, deaf children can be trained to focus and rely more on this visual cue.

#### 2.2.3. Cued Speech

For some children, it will be helpful to access extra visual cues to support the discrimination of speech sounds and complement lip-reading [24]. Cued speech is a technique in which the speaker codes syllables of speech; hand-shapes are used to code consonants and hand-placements around the face code vowels [19,25]. Cued speech takes approximately fifteen to twenty hours to learn, and can be mastered in less than three months, making it easily accessible to most parents.

#### 2.2.4. Coded Language

Another visual technique that can be used with deaf children is coded language, in which every word and morpheme orally produced by the speaker is represented by a gesture, such as Signed Exact English (SEE). Contrary to sign language, coded language is designed to support oral language and follows the oral grammar; it is essentially a simultaneous signed version of oral language. It can be especially useful for children who have difficulties perceiving small words and morphemes since they become visually apparent.

#### 2.2.5. Speech’s Natural Dynamic (Dynamique Naturelle De La Parole; DNP)

DNP, a multisensorial rehabilitative technique, which includes movement and rhythm, is sometimes used with deaf and hard-of-hearing children, mostly in French-speaking countries [26]. Clinicians and children make large movements representing syllables to help children know how to place their articulators and integrate the placements. Rhythm is also used to help children with the suprasegmental aspects of speech (e.g., intensity, length). Another important aspect of DNP is that learning to speak must be fun.

#### 2.2.6. Other Communication Techniques 

Other communication techniques can be explored, especially when the use oral or sign language is not sufficient to support communication. Natural gestures, drawing, writing or pictograms can help children to communicate. 

Since every child and family have different needs and objectives, clinicians can choose which techniques are adequate depending on each family particularities (such as preferences and expectations, region of residence, access to services, available education alternatives). It should be kept in mind that the choice of one or many techniques is flexible and can be (should be) adjusted during the rehabilitation process. More, as noted by Berland [27],

“… the communication between hearing families and their implanted deaf children evolves along a bipolar continuum whose two ends are oral communication alone vs a visual-gestural mode of communication alone [28]. In our population indeed, some families use the oral alone (but very often including some mimogestuality), others not using “official” sign language create conventional “house” or “family” signs [28], still others offer their child a spoken and signed linguistic environment (in our case, a “more or less signed”), others trained to cued speech use it more or less systematically, and more or less fluidly [29]. Oral language is thus the primary language used in all families (as found in hearing families of deaf children, [30]), but the target language offered by these families is very variable, and in fact, not so clear cut that it appears …”

### 2.3. Evidence of Efficacy

While the choice of any approach or technique should be based on the patient’s general portrait, including auditory abilities, presence of other disabilities, age at implantation and duration of deafness, residual hearing, hearing device performance, patient’s needs and preferences, and the expertise of the clinician, an evidence based-practice must also consider evidence of efficacy for these approaches. 

Four systematic reviews recently addressed this issue. Demers and Bergeron [31] investigated the efficiency of rehabilitation approaches for deaf and hard-of-hearing children. They noted that while more studies indicated that approaches with less use of signs seemed to be linked to a better speech and language development, high-level evidence was lacking to determine clearly which approach to choose with deaf and hard-of-hearing children. At the end, because of this lack of well-designed studies, the causal link cannot be established. Likely, the systematic review of Fitzpatrick et al. [32] compared oral only and oral + sign intervention used with children with hearing loss. They came to the conclusion that more data was needed to determine is the addition of signs was more efficient. A third systematic review [33] was interested in the efficiency of the AVT approach. The authors came to the conclusion that even if studies suggest a positive impact of AVT, more well-controlled studies must be completed to determine the efficiency of this approach. Finally, Brennan-Jones et al. [34] planned to investigate the same approach but could not include any article due to the lack of well-designed studies.

At the end, all four systematic reviews came to the same conclusion: there is a lack of evidence to support the choice of a particular and specific approach with children with hearing impairment. Because there is not clear evidence that an approach leads to a better oral development, the prioritized approach differs depending on the rehabilitation center. Some prefer a bimodal approach, arguing that it prevents language deprivation by implanting a communication mode before access to the sound of speech. Others prefer an oral-only approach, arguing that signs could delay oral development and reduce the emphasis put on oral language. As recommended by the Joint Committee on Infant Hearing [35], clinicians should have a flexible approach and offer families different communications options based on the particularities of the patient, rather than offer one unique rehabilitation approach. Also, parents could benefit of an access to deaf people and Deaf culture during their decision-making process, and even after if needed. Finally, when the child is old enough, his opinion should be taken into account in order to respect his right.

Contrary to rehabilitation approaches, scientific evidence concerning rehabilitation techniques is less developed. Rayes, Al-Malky and Vickers [22] made a systematic review about the effectiveness of auditory training with implanted children on many abilities, including auditory and speech skills. They observed that children who received auditory training showed improvement in their skills, and that these improvements could transfer to other skills. However, stronger evidence is needed to support those observations. In a critical review of the evidence, Brouns, Refaie and Pryce [36] showed that although the preliminary evidence indicates an improvement gained from auditory training in adult rehabilitation, the treatment effect size was modest; there remains a lack of large-sample RCTs on this issue. Studies on lipreading mainly focused on the development of reading abilities. Many studies suggest that lipreading skills would be a predictor of reading abilities since it would help the development of phonological representation of words [37,38,39,40]. Other studies suggest that cued speech would also help to develop reading abilities, but also phonological awareness skills and phonological skills [41,42,43,44].

## 3. Clinical Rehabilitation Care of the Deaf and Hard-of-Hearing Child

As stated before, the majority of parents of deaf and hard-of-hearing children want their child to communicate with their hearing families, preferably orally, in order to develop their linguistic and social competence. As a result, the development of optimal hearing skills is frequently at the center of early rehabilitation interventions for the deaf and hard-of-hearing child. Thus, auditory training is the foundation on which spoken language-based approaches sit. Indeed, speech therapy aimed at the development of communication including oral language development in children cannot be optimal without the use of auditory skills. These hearing skills also contribute to the development of written language. While limitations of older hearing technologies often hampered the integration of auditory training into speech therapy, hearing perception with newer technologies have re-emphasized the importance of hearing stimulation. Thus, regardless of the approach chosen (AVT, aural–oral or total communication), auditory training is considered as an integral part of the rehabilitation program for deaf and hard-of-hearing children. Auditory training can take various forms depending on the nature of the hearing device, on the child’s age and environment, or the organization responsible for providing services.

### 3.1. Children Using Hearing Aids

In Quebec, a range of services are available to children with mild to severe hearing loss who use hearing aids, including hearing deficit and auditory skills assessment, evaluation of hearing aids’ performance, recommendation of additional assistive devices, and auditory training. All these interventions are aimed for optimal listening based on constant use of well-fitted hearing aids in realistic listening conditions. Immersive rehabilitative technologies, where common sound experiences in terms of noise type, incoming directions, signal to noise ratios and temporal dynamics, are introduced to recreate the realistic daily conditions needed for assessment and rehabilitative therapies [45].

Typically, once the diagnosis of hearing loss is established, the child is enrolled in a rehabilitation program where the hearing assessment is extended to confirm the degree of severity and specify the hearing aids’ adjustment parameters. An evaluation of the prosthetic performance is performed to validate these parameters. These assessments can be carried out in professional dyad to ensure the child’s full collaboration and thus, an optimal evaluation of the auditory portrait.

This assessment also specifies the child’s auditory skills. Depending on the mastering of these skills, auditory training may be offered. This intervention can take the form of blocks of weekly individual therapies where the child is encouraged through various play activities to develop his listening skills. These blocks of therapies vary in duration according to the child’s needs and pace of progression. Typically, these therapies are offered to children with more severe hearing deficits. However, some children with a lesser degree of hearing loss may also benefit when their language development differs markedly from developmental expectations.

Follow-up assessments can be offered at regular intervals to support regular use of hearing aids by ensuring that devices are still well-fitted to the child’s needs. The observations collected from these assessments, combined with observations on language development, support the decision process on the initiation, the continuation, or the termination of auditory training therapies. 

As the intervention perspective is based on social participation, awareness-raising activities can be offered to the family and to the child’s daily-living communication partners. These meetings typically address themes related to the child’s social integration, including the hearing loss’ impact, communication strategies, hearing aids’ manipulation and adaptations to limit background noise. These meetings aim to empower the child’s communicators on constant use of hearing aids and language stimulation.

For school-age children, rehabilitation aims first and foremost to set optimal listening conditions and to develop children’s autonomy in the use of their hearing aids and complementary assistive devices (such as FM systems). A preferential placement of the child in class is also promoted.

### 3.2. Children Using Cochlear Implants

A cochlear implant is the most relevant technological choice in the presence of a severe to profound deafness. Given the severity of hearing loss, auditory training appears essential to the optimal development of auditory skills. Upstream, pre-implantation auditory training can help specify auditory skills with hearing aids and validate the candidacy for cochlear implantation.

Quebec’s rehabilitation program proposes an intensive functional rehabilitation period lasting several weeks, including daily therapies, after cochlear implantation. Auditory training is the cornerstone of these intensive rehabilitation programs. In addition, the daily recurrence of therapies enhances the parents’ involvement in the child’s rehabilitation process, promoting the learning of auditory stimulation skills and their generalization to everyday life, supporting the child’s auditory and language development. 

Such intensive functional rehabilitation program typically aims to develop sound awakening and the child’s curiosity for environmental sounds. Then, the goal focus on the discrimination of suprasegmental parameters; onomatopoeias are used in order to develop some ability to identify simple words drawn from the child’s receptive lexicon among a closed-set choice of stimuli. Once these steps are completed, the training involves the auditory recognition of words and utterances in an open choice set, and, ultimately, gets the child capable of understanding speech without visual cues.

Auditory training is based on documented learning mechanisms, such as those included in behaviorist models (choice of stimuli, induction of expected responses or behaviors and reinforcement). It involves playful activities and tangible/contingent reinforcements to stimulate and maintain the child’s attention and thus promote learning. 

Once the intensive program is completed, auditory training can be maintained on a regular basis (i.e., weekly therapy). The follow-up’ s duration is determined from various parameters (1) related to the child such as the child’s age, his level of progress in terms of auditory skills as well as language development or (2) related to human resources available for service providing. Follow-up can also be offered intermittently including break periods to allow learning consolidation and generalization.

For school-aged children, auditory training may vary depending on cochlear implant use’s duration, the level of improvement in auditory skills and the school environment. The care can take the form of a regular follow-up or close monitoring. As with children with hearing aids, auditory rehabilitation will focus on optimal listening conditions, including the recommendation and adjustment of assistive listening devices (such as FM systems).

### 3.3. Interprofessional Collaboration

**Figure 4:**
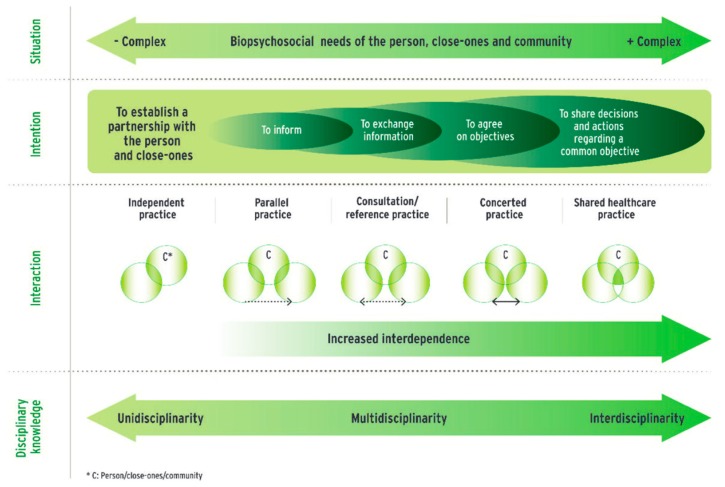
Continuum of Interprofessional Collaborative Practice in Health and Social Care [46]. + and − Complex indicators define the complexity of the needs of the person/close-ones/community and the biopsychosocial context in which these needs are to be met.

The clinical management of a child with deafness cannot be fully realized without the contribution of many health professionals. This multidisciplinary collaboration allows the attainment and generalization of auditory skills in different environments. According to the model of the Collaborative Network on Interprofessional Practices in Health and Social Services [46] (Figure 4), this multidisciplinary approach is not limited to a parallel practice, but rather takes the form of a practice of care and shared services where decisions and actions are collective and linked to a common goal, namely the functional social participation of the child. In addition to providing a more complete view of the child, this interdisciplinary practice allows for concerted action to support the child in the development of his or her hearing skills.

Interdisciplinarity manifests itself in different ways during the process of clinical management of the child with deafness. For example, as previously discussed, assessment is often done in a professional dyad typically involving the audiologist’s expertise and the specialized educator’s support in order to obtain an accurate portrait of the child’s auditory skills. The speech-language pathologist can also contribute to the evaluation by analyzing the child’s responses according to his phonological difficulties.

Audiologists, speech-language pathologists, psychologists and specialized educators can also cooperate to develop and maintain the assiduity of hearing aids’ use by supporting the child, his parents or environment with different strategies (parental guidance or complementary use of accessories in respect of the parents’ choices). Moreover, those health practitioners are usually in the front row to observe any sign of discomfort or malfunction of the equipment.

The speech language pathologist may be also involved in the selection of linguistic stimuli used for auditory training. By sharing a portrait of the child’s language skills, she can assist the audiologist in developing the child’s individualized treatment plan regarding auditory development.

Finally, as part of an interdisciplinary practice, all participants contribute to the auditory training program as multipliers of intervention goals, either by being another model of auditory stimulation, or by integrating auditory training objectives into their respective therapies. In addition, the specialized educator can accompany the child in his living environments where the child is challenged by real communication contexts (home, daycare, school, restaurant) and validate the transfer in the daily life of therapeutic gains in auditory, linguistic and psychosocial domains.

### 3.4. Variant Rehabilitation Care Models

The interdisciplinary practice model of rehabilitation for the deaf and hard-of-hearing child can be modeled by hearing health care systems. In France, for example, the professional offer can be different from what is found in America. Indeed, whereas in America therapeutic support is the responsibility of the speech-language pathologist and audiologist, and technological support is the responsibility of the audiologist and audioprosthetist, the scope of actions of speech-language pathologists in France in the field of deafness is broader given that the profession of audiologist does not exist. Therefore, the rehabilitation model differs according to the way professionals settle in.

Typically, in France, when a sensorineural hearing loss is diagnosed, care is provided for the child and his family. A speech and language therapy assessment is carried out to assess the impact of deafness on speech, oral language and learning abilities. This initial assessment, as well as the degree of deafness, the method of rehabilitation chosen by the family (use or not of technical aids, and if so, its type), the child’s mode of communication, the presence or not of associated disorders, the family’s educational and linguistic project will orient the therapeutic offer. If parents chose a hearing rehabilitation, it can be proposed before the age of 6 months in the event of severe and profound deafness. 

When hearing aids are considered, implementation (choice, adaptation, immediate effectiveness check) and follow-up (prosthetic education, checks, maintenance) are carried out by audioprosthesists, either in rehabilitation institutes or in specialized private practices. As mentioned by Toffin and Alis-Salamanca [46], speech therapy sessions should ideally precede fitting in order to prepare appointments and get to know the child before the hearing aids fitting. In this way, the speech-language pathologist can observe what the hearing aids brings in terms of hearing recovery and how the child appropriates it. 

When cochlear implant(s) is/are chosen, care is provided by cochlear implantation units, established within the ENT departments. Professionals setting the devices are either audioprosthetists or speech therapists. An audiometric evaluation is completed; a speech and language therapy assessment is systematically carried out.

Therapeutic support is then offered in dedicated facilities for children aged 0 to 3/6 years, taking into account the specific needs related to the deafness of young children (early education and family guidance): Centers for Early Medicosocial Action (CAMSP), Family Support and Early Education Services (SAFEP) or Specialized Education and Home Care Service (SESSAD). When these services do not exist on the regional territory, are saturated, or in addition to their support, professionals in private practice can also carry out this early intervention mission.

Depending on the setting, the work of the speech-language pathologists can differ on some points:

- The modalities of support for the child: in structure, the sessions are carried out in individual and/or small groups. Indeed, since services are most often specialized in early support, the children taken in may have similar needs, and peer group work is encouraged. The groups can be led by the speech therapist alone or with another professional (e.g., psychologist, psychomotor therapist, etc.). In the liberal exercise, work is most often carried out on an individual basis.
- The type of follow-up of the child and his family: in a structure, multidisciplinary medical, para-medical and educational support is provided. Physician, paediatric nurse, psychologist, speech therapist, psychomotor therapist, specialized teacher, specialized educator, early childhood educator, sign language educator, cued speech coder and social worker are all professions that generally compose these teams. The technical platform may differ according to the institutions. At the end of an evaluation period, a personalized child project is carried out in collaboration with the entire team and the family. Depending on the identified needs of the child and his family, individual or small group rehabilitation sessions are offered: discovery of the sound environment, awakening and development, socialization, speech therapy, psychomotricity, educational workshops. In private practice, the professional sometimes carries the therapeutic project alone. If other liberal professionals follow the child, he can link with them, but collaboration is more complex. Moreover, early diagnosis implies that the speech therapist can welcome deaf babies from 3 to 4 months of age into his private practice. This change from the more traditional private practice work and require the speech and language therapist to be trained in early education [47].

As the child grows up, the way in which the child attends school and the type of therapeutic support are often linked. Several modes of schooling are possible, and speech and language therapy rehabilitation approaches will depend on the child’s needs, but also on the speech and language therapist’s experience in the rehabilitation of deafness and/or in training with specific tools.

When the deaf child is able to attend regular schooling, he or she can be enrolled either in individual school integration with hearing peers with or without human assistance for integration in class (Special needs teaching assistants, cued speech coder, French Sign Language (Langue des Signes Française, LSF) interpreter) or in a bilingual school (LSF/written french, still poorly developed throughout the French territory). This decision is based on the chosen communication approach, and on the local availability of educational and therapeutic services supporting this choice. In any case, and in order to take into account the particularities of each child, flexibility in educational programming is essential [48] and personal adaptations of the supports are necessary.

Outside the classroom, when the family desires that the child develop oral language, and the child uses either conventional hearing aids or cochlear implant(s), a pluri-weekly rehabilitation follow-up in speech therapy is offered. For the majority of children, this support is provided from private practice. Following the assessment session, the speech and language therapist defines areas of work focused on explicit learning of speech and oral language: metaphonological skills, lexicon, syntax, communication skills... Language acquisition being considered in its multimodal aspects, the speech therapist will be able to rely on the child’s visual or even multi-sensory perceptions so that the child can grasp the most suitable modality for him or her to enter the language: lip reading, cued speech, Signed Exact French, verbo-tonal method, DNP/ “natural speech dynamics method”, etc. This learning does not exclude the acquisition of sign language whose lexicon and grammatical structure are different [49]. “Among all these approaches, it is a question of choosing the one(s) that best suits the deaf child” [50]. On the other hand, since LSF, like cued speech or Signed Exact French, requires learning, the speech-language pathologist must also support parents in their daily communication. This learning and its daily use can be more or less early depending on the families. If difficulties in entering written language are observed, the speech and language therapist will also support the child to develop its acquisition.

A school monitoring team made up of the different professionals working with the child (referent teacher, the child’s teachers, health professionals and social service professionals if needed) meets once a year with the family to create a link and oversees the implementation of the Personalized Schooling Project. The speech-language pathologist therefore participates when he is available, this being done on a voluntary basis.

When parents have chosen a visuogestual approach, the speech therapist in private practice can be solicited when difficulties in entering written language appear. The speech-language pathologist can also be involved if the child develops cross-disciplinary skills disorders (pragmatic, implicit, mathematical reasoning, visual memory and attention, executive functions). The main difficulty for families is then to find a speech-language pathologist who is familiar with the French Sign Language.

The speech and language therapist may observe that the gap between acquisition and the norm is increasing, or that other difficulties are emerging. In this case, he can request additional assessments in private practice or with teams specialized in developmental delays. The mission of professionals on these platforms is to assess complex disorders in a multidisciplinary way. When the deaf child presents specific developmental or learning difficulties which are more global, but which remain compatible with regular schooling, the speech therapy follow-up in private practice will be replaced by support within a medico-social service. In the field of hearing impairment, these are SSEFIS (Support Services for Family Education and School Integration) or SESSAD DA (special education and home care services specializing in hearing loss). This service is composed of a multidisciplinary team of professionals from the specialized or health field, who travel to intervene (care, rehabilitation or awareness-raising measures) in places where the child evolves (daycare center, school, leisure center, home, grandparents place...). They can also offer groups times within the service for children and/or families. 

Given the dearth of speech and language therapists in the salaried sector [51,52], with 31% of unfilled positions in health and medico-social services [53], these services are increasingly turning to speech therapists in private practice to provide care. This raises issues, as private practice professionals are themselves saturated and do not have the expertise that an employed speech and language therapist has been able to acquire in this specific field. Moreover, they do not benefit from the multidisciplinary perspective provided by the team and often cannot be present when individualized support projects are written.

If the child’s difficulties are incompatible with regular schooling, other education alternatives can be considered. Deaf and hard-of-hearing children or adolescents can be enrolled in a special education classroom (small school units), where they receive an education adapted to their difficulties [54]. They are present in ordinary schools, so that children can join a regular education classroom at certain times which are fitting for them, having thus inclusive times in ordinary classes and/or on playtimes. Depending on the units, speech and language therapy support is either provided by a specific multidisciplinary team or by professionals on a private practice.

When the child has a more severe disability, with associated handicaps of varying degrees (multihandicap or polyhandicap), a global, intensive and multidisciplinary care may be required. In France, in fifteen 20–25% of cases, other impairments may be associated with deafness [55]. These impairments are diverse, both in their nature and in their manifestations. The combination of impairments may defeat the usual methods of intervention. The problem of care becomes complicated and requires very specialized skills. In this case, a referral to a specialized medico-social institution (internally) is offered. Then, many professionals revolve around the deaf child and participate in his development: psychologist, psychomotor therapist, speech therapist, physiotherapist, occupational therapist, specialized teacher, educators, ENT doctor, social worker, audioprosthetist…The pooling of knowledge and regular consultation between all stakeholders is essential to adapt the support of children as closely as possible to their specific needs. When the family consents, members from the Deaf community can be part of the long term follow-up process; this could facilitate reflections on the identity and work future issues that the child will eventually face later in his life.

## 4. Prospects for the Future

New technologies have taken much place in the field of hearing health care and are an important vector of change in services. On one part, auditory technology have been largely influenced by the development of cochlear implants in the late 1980s and the rapid evolution of their processing algorithms. Cochlear implants profoundly changed the delivery of speech and language services for deaf and hard-of-hearing children who benefit from it. Indeed, there is a before and an after cochlear implant era. Moreover, early diagnosis has led to early intervention and support, including early cochlear implantation. Cochlear implantation is then often considered as a mean to access oral language, but also as the technical assistance that will facilitate the child’s entry into regular school when they do not have a developmental delay or significant associated disorders [56,57], an option desired by the majority of the implanted children’s parents [58]. However, even with the more recent versions of devices and “whatever future technological developments may bring, it is important to keep in mind that a CI does not restore normal hearing” [59]. On another part, visual and interactive innovations are also developing and contribute to the daily lives of deaf children/teenagers, schooling [60] and their speech and language care: services at a distance from translation of videos either in text or in sign language, virtual signers, pedagogical or rehabilitative applications, communication software, digital solutions to support learning and independence. Moreover, for children presenting more severe disabilities along communication difficulties, electronic communication aids (commonly called vocal synthesis) are modalities among the augmentative and alternative communication tools that can help to promote their personal development and their integration, both socially and professionally.

Those examples, among others, demonstrate that in recent decades, speech and language therapy work and intervention modalities have significantly evolved and will continue to evolve, following the experience of therapists, knowledge transfer from research, and technological and societal changes. Speech-language pathologists are therefore required to continuously train in order to keep up with the latest developments; student training should consider current psycholinguistic and neurolinguistic theories of child language acquisition (oral and signed), up-to-date theories and practices or oral rehabilitation, current auditory technologies (hearing aids and cochlear implants) and how they can be integrated into the deaf and hard-of-hearing child’s everyday life, and the potential contribution of deaf community resources. Moreover, changes in policies on support for children with special needs will also lead to forms of specialization, particularly with regard to the treatment of the most severe disabilities. Nevertheless, at the end of the day, professionals should never lose sight of the fact that the social participation of deaf and hard-of-hearing children is the targeted outcome and that communication is a key factor of this outcome, and communication is grounded on language development. Thus, regardless of the therapeutic approach, oral and/or signed based, language deprivation needs to be limited if not avoided.
